# Relationship between quadriceps muscle architecture and lower limb strength and physical function in older adults community-dwelling individuals: a cross-sectional study

**DOI:** 10.3389/fpubh.2024.1398424

**Published:** 2024-06-07

**Authors:** Fahri Safa Cinarli, Hilal Er Ulubaba, Ozan Ucar, Deniz Can Kilinc, Rukiye Ciftci, Raci Karayigit, Monira I Aldhahi, Sameer Badri Al-Mhanna, Mehmet Gülü

**Affiliations:** ^1^Department of Coaching Education, Faculty of Sport Sciences, Inonu University, Malatya, Türkiye; ^2^Department of Radiology, Yesilyurt Hasan Calik State Hospital, Malatya, Türkiye; ^3^Department of Anatomy, Faculty of Medicine, Inonu University, Malatya, Türkiye; ^4^Department of Coaching Education, Faculty of Sport Sciences, Ankara University, Ankara, Türkiye; ^5^Department of Rehabilitation Sciences, College of Health and Rehabilitation Sciences, Princess Nourah Bint Abdulrahman University (PNU), Riyadh, Saudi Arabia; ^6^Department of Physiology, School of Medical Sciences, Universiti Sains Malaysia, Kelantan, Malaysia; ^7^Center for Global Health Research, Saveetha Medical College and Hospitals, Saveetha Institute of Medical and Technical Sciences, Chennai, Tamil Nadu, India; ^8^Department of Sports Management, Faculty of Sport Sciences, Kirikkale University, Kırıkkale, Türkiye

**Keywords:** older adults, functional mobility, muscle architecture, ultrasound, physical function

## Abstract

**Background and objective:**

Factors related to muscle architecture may lead to functional limitations in activities of daily living in the older adults. This study aimed to investigate the relationship between quadriceps femoris (QF) architecture and physical function in older adults community-dwelling people.

**Methods:**

The study included 25 community-dwelling older adults participants aged over 60 years (14 women and 11 men) who were not engaged in regular physical activity. The rectus femoris (RF) and vastus intermedius (VI) muscle thicknesses as well as the RF cross-sectional area (CSA) were assessed using 2D ultrasonography. The 30 Seconds Chair Stand test (30sCST) and Timed Up and Go Test (TUG) were used to assess lower body muscle power and functional mobility, respectively.

**Results:**

The QF muscle architecture showed moderate and large correlations with the 30sCST (r range = 0.45–0.67, *p* < 0.05) and TUG (r range = 0.480–0.60, *p* < 0.05). RF thickness was a significant (*p* < 0.01) independent predictor of 30sCST (*R*^2^ = 0.45) and TUG (*R*^2^ = 0.36). VI thickness was a significant (*p* < 0.05) independent predictor of 30sCST (*R*^2^ = 0.20) and TUG (*R*^2^ = 0.231). RF CSA was a significant independent predictor of the 30sCST (*R*^2^ = 0.250, *p* < 0.05) and TUG (*R*^2^ = 0.27, *p* < 0.01). Multiple linear regression models explained 38% of the 30sCST variance and 30% of the TUG variance in the older adults group.

**Conclusion:**

Quadriceps muscle group directly affects basic activities of daily living in the older adults. Ultrasound measurements, which are non-invasive tools, are extremely valuable for understanding the limitations of activities of daily living in the older adults.

## 1 Introduction

Aging is an important life cycle that is slow, but profoundly affects the organism. It is well known that age-related complications have a negative impact on daily functional abilities and even limit simple life activities (1). Approximately that 15-35% of people in Europe aged 75 years and older need help in performing activities of daily living (2). As with many other structures and systems, some age-related changes and deterioration are observed in muscles (3, 4). Weakening of muscular structure is influenced by barriers to participation in physical activity and other factors (5–7). It is known that the structure of skeletal muscle changes with age, particularly due to the sarcopenic effect, and that there are changes in the architecture of skeletal muscle (8). It has been mentioned that about 21% of people over the age of 85 and even more are likely to suffer from sarcopenia, i.e., a loss of muscle strength and mass (9). In sarcopenia, which can be etiologically described as aging and physical inactivity, changes are also observed that can have a negative effect on movement kinematics, such as a decrease in the activation capacity of the motor units and an increase in the co-activation of the antagonist muscle (10, 11).

One evident physiological alteration observed in skeletal muscle tissue among older individuals is the presence of atrophic characteristics and notable decline in muscular strength (3). With the decline in skeletal muscle mass and the concurrent increase in adipose tissue, the daily execution of fundamental movement patterns may pose challenges in terms of management and execution (12). Apart from significant alterations such as a decline in visual acuity and vestibular function, falls can lead to injuries and even fatalities, particularly as a result of modifications in muscle tissue. Depending on age-related changes in muscle morphology, there is a reduction in the control of motor units and balance capabilities (13). The mortality risk resulting from falls is 1.48 times higher among individuals over the age of 80 years than among those below 80 years. Moreover, in 2019, the emergency room witnessed an influx of three million cases of fall-related injuries (14). A study by Zhang et al. (15) revealed that the age-standardized fall-related mortality rate was 10,438 per 100,000 individuals. Notably, the highest mortality rate was observed in individuals aged ≥85 years. World Health Organization Department for the Management of Noncommunicable Diseases and Injury (16) highlights that in low- and middle-income countries, the fall-related mortality rate exceeds 80%. Given these statistics, there is an increased impetus for researchers to delve into the determinants that affect the daily activities of the older adults (17, 18). Although numerous investigations have explored the impact of muscle morphology and structure on the athletic performance of healthy individuals (19–21), few studies have addressed how the architecture of skeletal muscles influences the ability of the older adults to perform daily activities (22).

Previous investigations have primarily focused on the association between isokinetic force and muscle structure rather than examining everyday movements such as sitting and walking (23, 24). As the present study specifically concentrates on activities of daily living, it has the potential to provide valuable insights into the healthy older adults population. We hypothesized that the quadriceps muscle architecture is significantly correlated with functional performance in community-dwelling older adults. Such findings would hold value for health care professionals and physiotherapists who frequently engage with the older adults. Our study aimed to elucidate the relationship between quadriceps muscle architecture and strength and functional capacity in the older adults.

## 2 Methods

### 2.1 Participants

Participants aged >60 years and those who did not engage in physical activity while being free from cardiovascular or respiratory disorders were included in the study. Exclusion criteria included any known muscular, neurological, metabolic, or inflammatory diseases, as well as uncontrolled hypertension or angina. The sample size was determined by a prior power analysis utilizing G-Power (version 3.1.9.3). Based on previous studies, the coefficient of determination (*R*^2^ = 0.35) was determined, which was considered a reasonable and conservative starting point for determining the sample size (25). The type I error (α) was 0.05, and the power (1-β) was 0.80 the bivariate normal model. The model indicated a minimum total sample size of 20 participants, but considering a miss rate of 25%, we decided to have a total of 25 elder people (14 women and 11 men; mean age ± standard deviation: 68.64 ± 6.92 years). The participant group consisted of healthy older adults living in a temperate climate and belonging to the middle-income group. None of the participants with similar physical activity levels had a history of long-term exercise. Therefore, the study group can be generalized as a sedentary, healthy, and older person. This study was approved by the Malatya Turgut Ozal University Clinical Research Ethics Committee (Protocol No:2023/13). The study was conducted in accordance with the principles of the Declaration of Helsinki. Informed consent forms were signed after the participants were informed of the study. Sociodemographic data were collected for each participant using a standardized form of patient information.

### 2.2 Study design

A cross-sectional study was conducted to investigate the relationship between muscle architecture and functional capacity in healthy older adults. The participants visited the hospital radiology clinic on two non-consecutive days. First, participants’ anthropometric and demographic data were collected. A familiarization session was also conducted to prevent learning effects during functional tests. 2-dimensional B-mode ultrasound images of the quadriceps muscle were obtained at the second visit. Although the participants invited to participate in the study were able to move functionally on their own, they were constantly monitored closely by two physiotherapists, two doctors, and a sports scientist to ensure that the measurements went smoothly. During the familiarization phase, a visual analysis was conducted, and the older adults also provided verbal feedback on the difficulty of the tests. The purpose of this procedure is to ensure the comfort and safety of the older adults. In this study, although there are devices such as magnetic resonance imaging and computed tomography that provide more detailed imaging capabilities than ultrasound, it can be said that the use of ultrasound is due to comfort, cost, and convenience, such as the ability of the patient to quickly change position during measurement. Functional tests were performed after the ultrasound measurements.

### 2.3 Procedures

#### 2.3.1 Muscle architecture measurements

The architecture was evaluated using a 2D real-time B-mode ultrasound device (Mindray DC-8 Exp) equipped with a high-frequency linear probe (3–13 MHz and 5 cm depth). The images were taken in the supine position with passive extension of the knee. The thicknesses of the rectus femoris (RF) and vastus intermedius (VI) were measured at the midpoint between the anterior superior iliac spine and proximal end of the patella. The probe was placed perpendicular to the skin without applying pressure, keeping it in a transverse position (26, 27). The cross-sectional area (CSA) of the RF was measured at three-fifths of the distance from the SIAS to the upper border of the patella. The probe was placed perpendicular to the longitudinal axis of the thigh on its upper side with the probe held in a transverse position (28). Contact gel was used to minimize the underlying soft tissue distortion. All measurements were performed by the same experienced sonographer, who was blinded to the participants.

Three ultrasound images were taken from each trial to increase reliability, and the average of the three values for each variable was used for statistical analysis. The intra-rater reliability for muscle architecture was evaluated using the intra-class correlation coefficient (ICC), coefficient of variance (CV), and standard error of mean (SEM).

#### 2.3.2 Lower body muscle strength

The 30 Seconds Chair Stand test (30sCST), developed by Rikli and Jones (29), is a reliable assessment tool for evaluating lower-extremity muscle strength in older individuals. This test has demonstrated efficacy in terms of both time efficiency and ease of use. Furthermore, it correlates with an individual’s ability to perform activities of daily living. This test measures how many times you can get up from a standard 45 cm highchair without arms within 30 s. Previous research has demonstrated that this test exhibits commendable repeatability and inter-rater reliability (30, 31). Prior to the measurements, the participants underwent a familiarization phase consisting of three repetitions, starting from a seated position with a neutral spine and feet planted flat on the ground, as detailed by Smith et al. (32). Following this phase, formal recording of the test was initiated with a start command, encouraging participants to complete the task as quickly as possible. A second attempt was made after a 5-min passive rest period, during which participants were instructed to remain seated.

#### 2.3.3 Physical function

Functional mobility was assessed using The Timed Up and Go Test (TUG) introduced by Podsiadlo and Richardson (33), which measures gait and dynamic balance. This test has been found to be associated with daily functional activities such as balance and walking speed (34, 35). This is a test to determine the time taken to stand up from a chair with an armrest with the command “Go” and to return from a flat surface of 3 m and sit down again. Previous studies have shown that it is a good and strong test in terms of test–retest and inter-rater reliability (36, 37). As it was established in advance that the type of chair influences performance outcomes, this study used a chair with an armrest and a seat height of 45 cm, which is within the recommended range (38). Before the official measurements, participants were given a trial session to familiarize themselves with the test. Participants were asked to sit comfortably on a chair and lean against the back of the chair. They were then asked to stand up and walk a 3 m distance in their usual way, turn around, walk back to the chair, and sit down again. The stopwatch was started when the patient’s buttocks were lifted from the seat, and the timing was stopped when the buttocks retouched the chair. Two trials were carried out and a 5-min passive rest was taken between trials.

### 2.4 Statistical analysis

Statistical analyses were performed using Statistical Package for the Social Sciences (version 24, IBM Corporation, NY, United States). Normality of data distribution was determined using the Shapiro–Wilk test. Pearson correlation was calculated between the muscle architecture and the results of the functional tests, and the variables were expressed as values with a 95% confidence interval (CI). Partial correlations were also analyzed considering age, gender, and BMI, and the variables were expressed. The correlation was interpreted as follows: an r between 0 and 0.3, was considered small; 0.31–0.49, moderate; 0.5–0.69, large; 0.7–0.89, very large; and 0.9–1, near perfect for predicting the relationship (39). If a correlation was significant, the independent parameter was entered into multiple linear regression. Variance inflation factor (VIF) and tolerance were used to examine the multicollinearity of variables in the model. If the largest VIF is >10 and the tolerance is <0.2, multicollinearity exists. Moreover, the intra-rater reliability for muscle architecture was evaluated using the intra-class correlation coefficient (ICC), coefficient of variance (CV), and standard error of mean (SEM). All data are presented as mean ± standard deviation with 95% confidence interval. The statistical significance level was set at alpha level of 0.05.

## 3 Results

Intra-rater reliability for muscle architecture showed that ICCs for RF thickness, VI thickness, and RF CSA were 0.987, 0.991, and 0.989, respectively. The mean CVs and SEMs of rectus femoris thickness, vastus intermedius thickness, and rectus femoris cross-sectional area were 2.09% and 0.06 cm and 6.5% and 0.65 cm and 3.1% and 0.30 cm^2^, respectively. Descriptive data for demographic, functional performance, and muscle architecture measures are shown in Table 1.

**Table 1:** Descriptive statistics for the demographic, muscle architecture and functional measures (*n* = 25).

Variables	Mean	SD	95% CI
Age (yr)	68.64	6.92	65.78–71.49
Height (m)	1.65	0.08	1.61–1.68
Weight (kg)	72.64	10.37	68.35–76.92
BMI (kg^.^m^−2^)	27.13	5.01	25.26–29.20
RF thickness (cm)	1.38	0.31	1.26–1.51
VI thickness (cm)	1.20	0.33	1.07–1.34
RF CSA (cm^2^)	5.33	1.51	4.70–5.95
30sCST (rep)	11.28	4.16	9.56–12.99
TUG (s)	10.22	3.49	8.78–11.66

BMI, body mass index; RF, rectus femoris; VI, vastus intermedius; RF CSA, rectus femoris cross-sectional area; 30sCST, 30 s chair stand test; TUG, timed up and go test.

The Pearson and partial correlations between the quadriceps muscle architecture and functional tests are shown in Table 2. Based on bivariate and partial correlation analyses, after adjustment for age, gender and BMI, there was a positive linear correlation between the 30sCST and the thicknesses of RF (*r* = 0.672, *p* = 0.001; partial *r* = 0.691, *p* < 0.001) and VI (*r* = 0. 0.452, *p* = 0.023; partial *r* = 0.550, *p* = 0.008), and RF CSA (*r* = 0.500, *p* = 0.011; partial *r* = 0.510, *p* = 0.015). In addition, a negative linear correlation was found between the TUG test and RF thickness (*r* = −0.603, *p* = 0.001; partial *r* = −0.630, *p* = 0.002), VI (*r* = −0.480, *p* = 0.015; partial *r* = −0.650, *p* = 0.001), and RF CSA (*r* = −0.519, *p* = 0.008; partial *r* = −0.554, *p* = 0.007) (Table 2).

**Table 2:** Matrix of the Pearson and partial correlation between muscle architecture parameters (RF, VI and CSA = cross sectional area) and functional tests (30sCST and TUG) with age, gender and BMI controlled (*n* = 25).

Variables	30sCST				TUG			
	*r*	*p*	partial r	*p*	*r*	*p*	partial r	*p*
RF thickness	0.672	0.001	0.691	<0.001	−0.603	0.001	−0.630	0.002
VI thickness	0.452	0.023	0.550	0.008	−0.480	0.015	−0.650	0.001
RF CSA	0.500	0.011	0.510	0.015	−0.519	0.008	−0.554	0.007

r, Pearson correlation; partial partial r, adjusted for age, gender and BMI; 30sCST, 30 s chair stand test; RF, rectus femoris; VI, vastus intermedius; CSA, cross-sectional area; TUG, timed up and go test.

**Figure 1:**
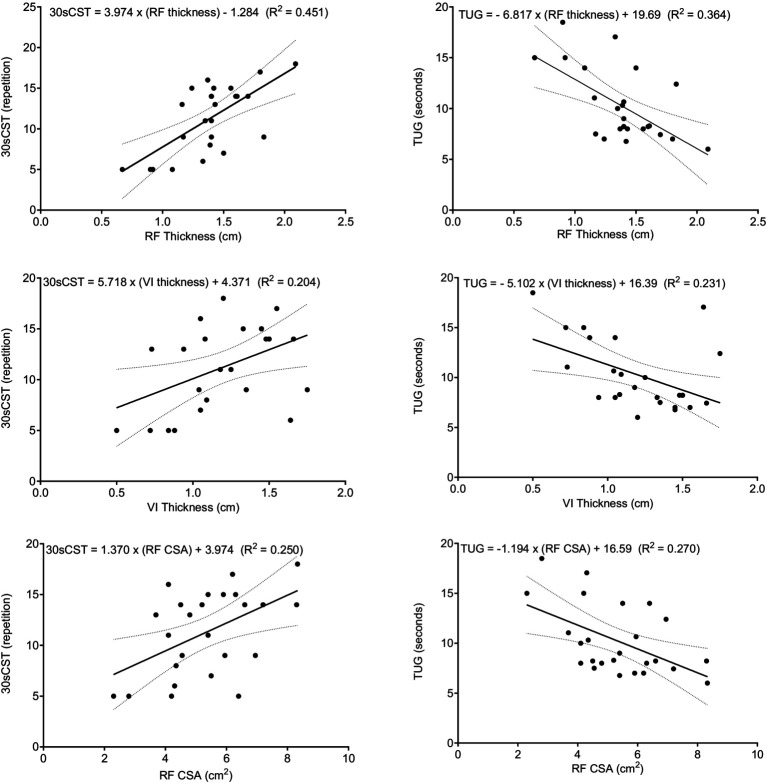
Scatter plots for muscle architecture parameters (RF, VI, and CSA), functional tests (30sCST and TUG), and linear regression lines with 95% CI. 30sCST, 30 s chair stand test; TUG, timed up-and-go test; RF, rectus femoris, VI, vastus intermedius, and CSA, cross-sectional area.

According to the results of the simple linear regression, RF thickness was the parameter that best explained the dependent variables and accounted for 45% of the variance in the 30sCST (F _(1,23)_ = 18.924, *p* < 0.001) and 36% in TUG (F _(1,23)_ = 13.136, *p* = 0.001). Conversely, VI thickness was responsible for 20% of the variance in the 30sCST (F _(1,23)_ = 5.901, *p* = 0.023) and 23% in TUG (F _(1,23)_ = 6.893, *p* = 0.015). The RF CSA also contributed as an independent factor, explaining 25% of the variance in 30sCST (F _(1,23)_ = 7.677, *p* = 0.011) and 27% in TUG (F _(1,23)_ = 8.489, *p* = 0.008) (Figure 1). In addition, the participants’ time decreased 6.81 s for each cm of RF thickness, 5.1 s for each cm of VI thickness and 1.2 s for each cm^2^ of RF CSA. Furthermore, repetition of the 30sCST was increased by 9.04 for each cm of RF thickness, 5.71 for each cm of VI thickness and 1.37 for each cm^2^ of RF CSA.

According to the collinearity statistics performed before the multiple regression model was created, there was no multicollinearity between the independent variables (tolerance and VIF range = 0.429–0.580 and 1.725–2.331, respectively). All muscles were included in the regression model and no control variables were used. As a result of the analysis, a significant regression model was found for 30sCST score (F _(3,21)_ = 5.820, *p* = 0.005). 38% (R^2^adj. = 0.376) of the variance in 30sCST repetition was explained by independent variables in the older adults (model for 30sCST = −1.514 + 8.300 × RF_thickness_ + 0.628 × VI_thickness_ + 0.095 × RF_CSA_). The regression model for the time of the TUG test was found to be statistically significant (F _(3,21)_ = 5.108, *p* = 0.030). Architectural measurements of the quadriceps femoris explained 30% of the TUG variance (*R*^2^adj. = 0.305) in the older adults (model for TUG = 20.294–4.652 × RF_thickness_ – 1.498 × VI_thickness_ – 0.338 × RF_CSA_) (Table 3).

**Table 3:** Multiple linear regression analyses of the prediction of functional measures from quadriceps muscle architecture in the older adults (*n* = 25).

Dependent variables	Independent variables	*β*	*t*	R^2^	Adj-R^2^	*F*	*p*
30sCST (rep)	RF thickness	0.61	2.50	0.45	0.38	5.82	0.005
	VI thickness	0.05	0.23				
	RF CSA	0.03	0.14				
TUG (s)	RF thickness	−0.41	−1.58	0.39	0.30	4.51	0.014
	VI thickness	−014	−0.63				
	RF CSA	−0.14	−0.59				

RF, rectus femoris; VI, vastus intermedius; RF CSA, rectus femoris cross-sectional area; 30sCST, 30 s chair stand test; TUG, timed up and go test.

## 4 Discussion

In this study, significant bivariate correlations were found between quadriceps muscle architecture in community-dwelling older adults individuals and basic activities of daily living, such as walking and rising from a chair. Additionally, these data show that the quadriceps muscle accounts for approximately 30–40% of the variance in physical functioning. The results provide promising evidence for future research exploring the role of muscle architecture in daily living activities. Muscle loss in old age is generally associated with inactivity, increased total fat, and decreased trophic hormone levels (40). All these rational mechanisms help to understand muscle architecture and the etiology of its negative impact on activities of daily living. In the context of these activities, Selva Raj et al. (22) have also examined the association between muscle wasting and functional limitations. Their findings indicated a negative linear correlation between lateral vastus thickness and combined rectus femoris-vastus intermedius thickness and performance in 6-m fast walking, timed standing, and walking (range *r* = 0.35–0.51, *p* < 0.05). Additionally, the muscles measured accounted for 27–39% of the variance in the Timed Up and Go test outcomes.

Saito et al. (41) conducted a study where a multiple regression model, which included age and gender along with the CSA of the quadriceps femoris, was found to account for 64% of the variance in performance on a sit-to-stand test. In our study, according to the results of the simple linear regression, the thickness of the RF explained 36–45% of the dependent variable. The VI thickness contributed to explaining 20–23% of the variance, while the RF CSA value accounted for 25–27%.

In a recent study conducted by Mateos-Angulo et al. (25), the prediction of rectus femoris muscle thickness in 12 older adults after five repetitions of the sit-to-stand test yielded results similar to ours, with an explained variance of approximately 35%. Wilhelm et al. (42) also observed a moderately significant correlation (*r* = 409, *p* < 0.05) between the 30sCST and quadriceps muscle thickness. Our findings align with previous research that has established a link between VI and RF thickness and 30 s of sit-to-stand, with correlation coefficients ranging from 0.452 to 0.672 and *p*-values less than 0.01. In contrast to our results, Nishihara et al. (43) did not find a significant association between rectus femoris and vastus intermedius thickness and TUG scores in men aged 65 and over 80 years. Similarly, Rech et al. (8) reported no significant association between quadriceps muscle thickness and the 30sCST in their study. However, they discovered a strong correlation between quadriceps muscle thickness and the usual walking speed (*r* = 0.509, *p* < 0.01). These discrepancies may be due to differences in the participants’ activity levels and previous injuries. When the profiles of participants in previous studies were examined, it was found that they were either physically active or did not mention this issue. There was also no explanation for the participants’ previous injuries. Therefore, it is difficult to draw conclusions as the participants’ profiles were not described in detail in previous studies.

A decrease in the number of parallel sarcomeres in skeletal muscle affects the physiological cross-sectional area, resulting in a decrease in force production (44). This situation may negatively affect the walking speed in the older adults by disrupting the contraction velocity of the muscle-tendon unit. In one study, walking speed and stride length were found to be related to vastus lateral (45). The maximum shortening rate in the older adults has been found to be at least 10% lower than that in young adults, but this difference may be greater in the older adults because of factors such as the extremely low isoform of type I heavy myosin chain, inhibition of agonist muscles, and excitation of antagonist muscles (4). These rational mechanisms, which relate to the changes that occur in skeletal muscles with age, explain why walking speed decreases in older people. This expectation supports the negative relationship between walking time and muscle thickness and RF CSA observed in our study.

Muscle atrophy and structural changes in the lower extremities are common, especially after 50 years of age (46). The medialis muscles of the right and left gastrocnemius, key components of the lower extremities, account for 15–32% of the variability in performance on the timed-up-and-go and five times sit-to-stand tests (47). The relationship between the thickness of the right and left gastrocnemius medialis muscles, one of the most important muscles of the lower extremities, to explain the results of the timed-up-and-go and the five times sit-to-stand test was 15–32% (47). In contrast, quadriceps strength, the largest muscle group in our body, was associated with stride length and walking speed (in older adults aged 60–88 years, *r* = 0.56, *p* < 0.001 and *r* = 0.51, *p* < 0.002, *n* = 34, respectively) (48). Furthermore, fall incidence has been inversely associated with quadriceps strength and directly with age in patients over 70 years (49). While falls in older adults are often linked to deficiencies in the visual, somatosensory, and vestibular systems (50), it is evident that alterations in the quadriceps musculature also play a role in fall etiology. This is supported by observations of diminished isometric and isokinetic performance accompanying losses in quadriceps mass and thickness (51, 52). In addition, RF and VI muscle thicknesses have been found to significantly influence the predictive models for peak knee extensor torque across various angular velocities, with the vastus lateralis also contributing, albeit to a lesser extent (22). In our study, the correlation coefficient values related to quadriceps muscle thickness showed that each unit increase could influence walking time by >5 s and the number of repetitions by >5. This emphasizes the important impact of clinical findings on daily life. Therefore, in our investigation, the measured thicknesses of the RF and VI within the quadriceps group were indicative of functional test outcomes, particularly those assessing concentric force generation, such as the action of rising from a seated position.

One of the limitations of this study was the lack of isometric and isokinetic testing procedures. The incorporation of a test modality with controlled angular velocity could provide valuable insights for researchers. However, given that the primary objective was to assess the impact of quadriceps muscle strength on everyday activities, the study was confined to the evaluation of free movement functions. Another limitation is that gender differences were not accounted for in the analysis, which could have influenced the outcomes. The potential for variance in muscle architecture and functional capacity between males and females exists, and this factor may play a significant role in the interpretation of the data. Future studies are required to explore these disparities in depth to understand better how gender-specific physiological differences may affect the relationship between muscle thickness, strength, and functional performance in the older adults. In addition, the one-time repetition of the functional tests in the study may represent a shortcoming in terms of the reliability of the test. To prevent this, it may be advisable to repeat the tests more than once, despite the familiarization time we spent with the participants, as variables such as excitement may come into play during official measurements. Nonetheless, the outcomes of this study contribute meaningful clinical insights pertinent to the non-exercising older adults male and female populations.

## 5 Conclusion

In summary, the quadriceps muscle significantly affects the functional abilities required for activities of daily living. Although the regression results emphasize the association between RF thickness and functional ability, VI thickness and RF CSA are also important explanatory factors. These results suggest that the architecture of this muscle group has the potential to be used to assess functional ability in older people. These data suggest that clinicians can obtain a picture of the functional status of healthy or sick older people by measuring the muscle architecture of the lower extremities using B-mode ultrasound.

## Data availability statement

The raw data supporting the conclusions of this article will be made available by the authors, without undue reservation.

## Ethics statement

The studies involving humans were approved by Malatya Turgut Ozal University Clinical Research Ethics Committee (Protocol No:2023/13). The studies were conducted in accordance with the local legislation and institutional requirements. The participants provided their written informed consent to participate in this study.

## Author contributions

FC: Conceptualization, Data curation, Formal analysis, Investigation, Methodology, Resources, Software, Writing – original draft, Writing – review & editing. HU: Writing – original draft, Writing – review & editing, Conceptualization, Data curation, Formal analysis, Investigation, Methodology. OU: Conceptualization, Formal analysis, Investigation, Methodology, Writing – original draft, Writing – review & editing. DK: Conceptualization, Data curation, Formal analysis, Investigation, Methodology, Writing – original draft, Writing – review & editing. RC: Conceptualization, Data curation, Formal analysis, Investigation, Methodology, Writing – original draft, Writing – review & editing. RK: Writing – original draft, Writing – review & editing. MA: Writing – original draft, Writing – review & editing, Funding acquisition. SA-M: Writing – original draft, Writing – review & editing. MG: Writing – original draft, Writing – review & editing, Supervision, Visualization.
